# Protocol of the PSYCHOTSH study: association between neonatal thyroid stimulating hormone concentration and intellectual, psychomotor and psychosocial development at 4–5 year of age: a retrospective cohort study

**DOI:** 10.1186/2049-3258-72-27

**Published:** 2014-08-18

**Authors:** Caroline Trumpff, Johan Vanderfaeillie, Nathalie Vercruysse, Jean De Schepper, Jean Tafforeau, Herman Van Oyen, Stefanie Vandevijvere

**Affiliations:** 1 Unit of Public Health and Surveillance, Scientific Institute of Public Health, Brussels, Belgium; 2 Faculté des Sciences Psychologiques et de l’Education, Université Libre de Bruxelles, Brussels, Belgium; 3 Faculty of Psychology and Educational Sciences, Vrije Universiteit Brussel, Brussels, Belgium; 4 Department of Paediatric Endocrinology, UZ Brussel, Brussels, Belgium

**Keywords:** Iodine deficiency, Thyroid stimulating hormone, Child development, Cognitive development, Psychomotor development, Psychosocial development

## Abstract

**Background:**

Several European countries, including Belgium, still suffer from mild iodine deficiency. Thyroid stimulating hormone (TSH) concentration in whole blood measured at birth has been proposed as an indicator of maternal iodine status during the last trimester of pregnancy. It has been shown that mild iodine deficiency during pregnancy may affect the neurodevelopment of the offspring. In several studies, elevated TSH levels at birth were associated with suboptimal cognitive and psychomotor outcomes among young children. This paper describes the protocol of the PSYCHOTSH study aiming to assess the association between neonatal TSH levels and intellectual, psychomotor and psychosocial development of 4–5 year old children. The results could lead to a reassessment of the recommended cut-off levels of 5 > mU/L used for monitoring iodine status of the population.

**Methods:**

In total, 380 Belgian 4–5 year old preschool children from Brussels and Wallonia with a neonatal blood spot TSH concentration between 0 and 15 mU/L are included in the study. For each sex and TSH-interval (0–1, 1–2, 2–3, 3–4, 4–5, 5–6, 6–7, 7–8, 8–9 and 9–15 mU/L), 19 newborns were randomly selected from all newborns screened by the neonatal screening centre in Brussels in 2008–2009. Infants with congenital hypothyroidism, low birth weight and prematurity were excluded from the study. Neonatal TSH concentration was measured by the Autodelphia method in dried blood spots, collected by heel stick on filter paper 3 to 5 days after birth. Cognitive abilities and psychomotor development are assessed using the Wechsler Preschool and Primary Scale of Intelligence - third edition - and the Charlop-Atwell Scale of Motor coordination. Psychosocial development is measured using the Child Behaviour Check List for age 1½ to 5 years old. In addition, several socioeconomic, parental and child confounding factors are assessed.

**Conclusions:**

This study aims to clarify the effect of mild iodine deficiency during pregnancy on the neurodevelopment of the offspring. Therefore, the results may have important implications for future public health recommendations, policies and practices in food supplementation. In addition, the results may have implications for the use of neonatal TSH screening results for monitoring the population iodine status and may lead to the definition of new TSH cut-offs for determination of the severity of iodine status and for practical use in data reporting by neonatal screening centres.

## Background

According to the World Health Organization (WHO), iodine deficiency is the main cause of preventable brain damage [1]. Iodine is important for the production of thyroid hormones (TH), which are essential for the development of the central nervous system [2]. Severe iodine deficiency may lead to perinatal mortality and mental retardation [3]. Maternal iodine deficiency during pregnancy, even at mild to moderate levels, may affect the neurodevelopment of the offspring [4]. Median urinary concentration is used to define classification of the levels of iodine intake as shown in Table 1.

**Table 1:** Median UI in school aged children: indicator of iodine nutrition

**Median UI (μg/l)**	**Iodine nutrition**
<20	Severe iodine deficiency
20-49	Moderate iodine deficiency
50-99	Mild iodine deficiency
100-199	Iodine sufficiency
200-299	Iodine intake more than adequate
>300	Iodine excess

*Source*: WHO, 2004 [1].

Iodine deficiency is defined through three levels of severity: mild, moderate or severe. Severe iodine deficiency has been observed mainly in Central Africa and Asia and is considered to be disappeared in Europe. However, several European countries, including Belgium, still suffer from mild iodine deficiency (MID) despite implementation of salt iodization programmes as national measures to supress iodine deficiency [5-8]. From 2003 to 2007, the number of European countries which are mildly iodine deficient decreased from 23 to 14 [9] showing that iodine deficiency remains a problem in Europe. Iodine deficiency even re-emerged in countries previously iodine sufficient such as the UK [10]. With respect to Belgium, recent studies found that school aged children were iodine sufficient while women at childbearing age and pregnant women had MID [7,8]. It is a matter of concern as MID during pregnancy could lead to suboptimal cognitive and psychomotor outcomes in the offspring [4].

**Figure 1:**
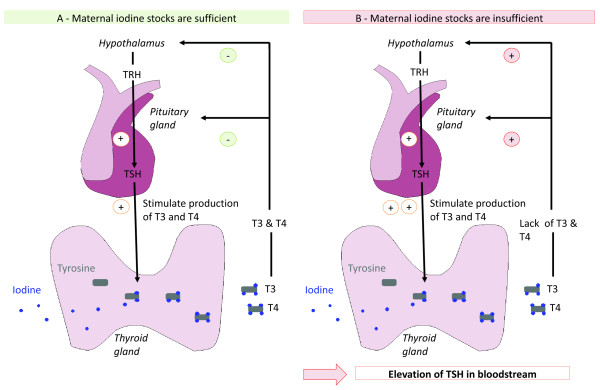
**Neonatal thyroid stimulating hormone concentration as indicator of maternal iodine deficiency.***Legend*: In the thyroid gland, thyroid hormones (TH) T3 and T4 are produced from association of tyrosine and iodine. Liberation of thyroid stimulating hormones (TSH) stimulates TH production and secretion. The lack of TH in the bloodstream leads to the liberation of thyrotropin-releasing hormone (TRH) from the hypothalamus. TRH stimulates the liberation of TSH by the pituitary gland. **A** - In situation of maternal iodine sufficiency, iodine stocks are sufficient to produce adapted amount of TH and TSH release is inhibited by negative feedback inhibition. **B** - In situation of maternal iodine deficiency, iodine stocks are insufficient to produce adapted quantity of TH and TSH release is maintained leading to increased TSH concentration in bloodstream.

Thyroid stimulating hormone (TSH) concentration in whole blood measured at birth has been proposed as an indicator of maternal iodine status during the last trimester of pregnancy [11]. TSH controls and stimulates the production of TH. In order to maintain circulating TH levels within the required range, TSH is secreted. If iodine stores are insufficient to produce TH, TSH concentration increases. Figure 1 illustrates the change in neonatal TSH concentration in case of maternal iodine sufficiency or deficiency during pregnancy.

Several studies showed that elevated TSH levels after birth were associated with suboptimal cognitive and psychomotor outcomes [12-15]. Though, it is hard to claim that the observed impairments in cognitive and/or psychomotor functioning are a direct consequence of MID during gestation. An elevated TSH level at birth can be caused by several factors [16-27] and some of them affect both TSH levels and IQ in childhood [14,23,28-59]. Further studies are needed to evaluate the association between elevated TSH at birth and intellectual, psychomotor and psychosocial development of preschool children taking into account confounding factors.

In order to evaluate the efficiency of national programmes implemented to suppress iodine deficiency, adapted surveillance of the iodine status of the population is important. To monitor the iodine status of a population, median urinary iodine excretion, thyroid size, neonatal TSH concentration in blood and thyroglobulin concentration in blood can be used as indicators [60-63].

In most developed countries, neonatal screening programmes for congenital hypothyroidism are organised allowing early detection and treatment of children with thyroid hormone therapy [64]. When neonatal screening programmes are set up in a country, the WHO recommends the use of TSH results to monitor iodine status in that population [60].

The percentage of neonatal TSH screening results greater than 5 mU/L can be used to define the iodine status of a population (see Table 2) as follows: a frequency below 3% indicates iodine sufficiency, a frequency of 3–19.9% indicates mild iodine deficiency, a frequency of 20–39.9% indicates moderate deficiency and a frequency above 40% indicates iodine deficiency respectively [27,60,61].

**Table 2:** Percentage of neonatal TSH screening results >5 mU/L at screening: indicator of iodine nutrition

**Percentage of neonatal TSH > 5 mU/L**	**Iodine nutrition**
0-3%	Iodine sufficiency
3-19.9%	Mild iodine deficiency
20 à 39,9%	Moderate iodine deficiency
≥ 40%	Severe iodine deficiency

*Source*: WHO, 1994 [60].

In several studies, the neonatal TSH results have been used to assess the iodine status of the population [65-68]. However the cut-off of 5 mU/L recommended by the WHO has been criticized [69-71]. In addition, a percentage below 3% of the TSH results greater than 5 mU/L was found in populations with MID [27,72] failing to detect MID in those population. Since the proposed cut-off of percentage of TSH screening results >5 mU/L below 3% is not sensitive enough to detect MID, it should be reassessed.

### Research objectives

The purpose of the PSYCHOTSH study is to assess the relationship between neonatal TSH levels and intellectual, psychomotor and psychosocial development of children aged 4–5 years old. It is hypothesized that children with a neonatal TSH level (at day 3 to 5) in the highest quintile, used as marker of lower intrauterine iodine supply, have a higher risk to develop psychomotor, cognitive as well as behavioural problems. In addition, the study aims to reassess the cut-off of 5 mU/L of neonatal TSH concentration in whole blood proposed by the WHO [60] to monitor iodine status in a population. The relationship found between TSH level and children’s intellectual, psychomotor and psychosocial development will be used to define a cut-off to indicate iodine deficiency using ROC curve analysis.

## Methods

### TSH determination

The children were selected from the total sample of neonates screened in 2008 and 2009 by the Brussels newborn screening centre for metabolic disorders (Laboratoire de Pédiatrie, Université Libre de Bruxelles, Brussels). Neonatal TSH level was measured in dried blood spots on filter paper collected by heel stick 3 to 5 days after birth. The measurement of TSH was performed using a time-resolved fluroimmunoassay (Autodelfia method) on filter paper [73].

### Subjects

A sample size of 315 children was determined based on a detection probability (or power) of 95%, a significance level alpha of 5% and a correlation factor between TSH and IQ of 0.2. An anticipated drop-out of 20% was taken into account. 380 children aged 4–5 years old with a neonatal TSH concentration in the range 0–15 mU/L were included in the study. Children were stratified by sex and by TSH level using a stratified sampling methodology. The details of the sample are shown in Table 3.

**Table 3:** Sample stratification of newborns by neonatal TSH level and by sex

**Neonatal TSH level mU/L**	**Boys**	**Girls**	**Total**
1-0	19	19	38
1-2	19	19	38
2-3	19	19	38
3-4	19	19	38
4-5	19	19	38
5-6	19	19	38
6-7	19	19	38
7-8	19	19	38
8-9	19	19	38
9-15	19	19	38
Total	190	190	380

For each sex and TSH-interval (0–1, 1–2, 2–3, 3–4, 4–5, 5–6, 6–7, 7–8, 8–9 and 9–15 mU/L) 19 newborns were selected randomly. In addition, the day of collection was taken into account. Exclusion criteria were: 1) neonatal TSH level of 15 mU/L or higher as this is the cut-off of TSH level for suspected congenital hypothyroidism, 2) prematurity (<37 weeks) and low birth weight (<2500 g) as this may induce TSH elevation at birth and alteration of neurodevelopment, 3) neurological disease of the children and 4) non-French speaking children (in order to perform psychological testing in the children’s mother tongue). For each selected infant, 3 replacements (with same TSH level and sex) were randomly selected, in case of refusals or non-contactable individuals.

### Approval by Ethical Committee for human subjects and by Privacy Commission

A written informed consent is obtained from the parents before the start of the procedure (i.e. home visit). The study design, the data collection procedures, the consent form and the invitation letter were approved by the Ethical Committee of the Erasme hospital (Université Libre de Bruxelles, Brussels) in accordance with the Code of Ethics of the World Medical Association for experiments involving humans (Declaration of Helsinki). The study was also approved by the Belgian Privacy Commission.

### Psychological testing

Psychometric assessment is done by 7 trained clinical psychologists during a home visit. Psychologists are blinded for the neonatal TSH levels of the selected children. Before test administration, the parents are asked to provide a quiet room and leave the psychologist alone with the child. If the child does not feel comfortable being left alone with the psychologist, one adult may stay in the room with the child, outside his or her field of view and without interrupting the psychologist.

Cognitive development is assessed using age-appropriate cognitive tests, the French version of the Wechsler Preschool and Primary Scale of Intelligence - third edition (WPPSI-III) [74,75]. The WPPSI-III is a test administered individually and allows to measure intelligence of children aged 2 years 6 months to 7 years 3 months; it is divided into 2 age groups: 2 years 6 months to 3 years 11 months old and 4 years to 7 years 3 months old. The test contains 14 subtests: 7 verbal tests, 5 performance tests and 2 processing speed tests. These subtests allow calculating a full scale Intelligence Quotient (IQ), a verbal IQ, a performance IQ, a processing speed quotient and an optional score of general language knowledge.

Psychomotor development is assessed using the French adapted version of the Charlop-Atwell Scale of Motor Coordination [76,77]. This individually administered test evaluates gross motor coordination abilities of children of 3½ to 6 years old using 6 subtests. The scale contains a subjective rating (based on quality of performance) in addition to an objective subtest rating (based on accuracy of performance).

Psychosocial development is evaluated using the French version of the Child Behaviour Check List (CBCL) for ages 1½ to 5 years old [78]. This test allows obtaining 3 scores: a total problem score, a score on internalizing problems and a score on externalizing problems. Internalizing problems represent problems of the self, like withdrawal, anxiety or depression. Externalizing problems are problems with others, problems of conflicts or problems with authority, like aggressive behaviour or attention problems. The total problem score includes these 2 scores plus a score on sleeping problems and a score on “other problems”. In order to calculate the scores, the Windows Software Assessment Data Manager is used [78].

### Children anthropometric measurements

Next to psychometric assessment, the actual weight, height and head circumference of the child are measured using SECA 815 or SECA 804 weight scales, SECA 214 stadiometers and SECA 212 flexible measuring tapes.

### Children urinary iodine concentration

A sample of urine is collected from the child during the home visit. The aim is to determine the current iodine status among the children included in the study. All urine samples are frozen and kept at -80°C until analysis. Analyses of the samples are performed at the Erasme Hospital. Urinary iodine excretion is measured using a modification of the Sandelle-Kolthoff reaction with spectrophotometric detection [79].

### Data collection of covariates, effect modifiers and descriptive variables

The variables collected in this study as covariates, effect modifiers and descriptive variables are shown in Table 4.

**Table 4:** Covariates, effects modifiers and descriptive variables of the PSYCHOTSH study

**Covariates**	**Effect modifiers**		**Descriptive variables**
Association of elevation of TSH and impaired	Elevation of TSH	Neurodevelopment and/or psychometric testing	
Neurodevelopment			
Foetus in utero exposure to:	Foetus in utero exposure to:	Child related factor:	Zip code of the house of the child
		Child bilinguism	
Iodine excess	Iodine containing drugs	Chronic disease of the child	Date of birth of the
TSH-receptor blocking antibodies from mothers with autoimmune thyroid disease		Attending nursery school	child, mother and father
			Height and weight of the child at birth
Antithyroid drugs	Exposition of the new-born during neonatal period:	Previous intellectual assessment	Actual height, weight of child, mother and father
		Child negative life events	
Contrast agents	Exposition to cold	Parenting stress	
Organochlorides	Surgical hypothermia	Dysfunctional parenting	Head circumference of the child
Lithium	Delivery by forceps extraction		
Cadmium			Household composition
Maternal smoking	TSH testing:		Nationality and origin of the child and the parents
Maternal alcohol consumption	Timing of blood sampling		
	TSH assay used	Parents related factors:	Maternal/paternal education and employment
		Level of education	Household incomes
		Household incomes	Marital status and custody
		Maternal age at birth	
		Parity	
Exposition of the new-born during neonatal period:			
Exposure to iodine-containing antiseptics		Gravidity	
Perinatal anoxia		Pre-pregnancy body mass index	
		Weight gain of the mother during pregnancy	
		Maternal diabetes during pregnancy	
		Maternal diabetes treatment during pregnancy	
		Maternal mental health disorders	
		Maternal poor social support	
		Marital discord	

Information about covariates and effect modifiers is gathered from the data provided by the ULB newborn screening centre for metabolic disorders. Additional information about pregnancy and about the period between birth and the home visit at 4–5 year old is collected using a self-report questionnaire filled in by the mother and from consultation of the health booklet of the child by the psychologist. In addition, the name of the gynaecologist is requested in the informed consent form in case additional information will be needed regarding the health of the mother during pregnancy.

Concerning pregnancy the following data are collected: exposure to iodine excess (use of iodine- containing antiseptics), intake of iodine-containing drugs, exposure to organochlorides, exposure to cadmium, lithium intake, thyroid disease of the mother, anti-thyroid drug intake, diabetes and treatment of diabetes, alcohol consumption and cigarette smoking, maternal age at birth, reproductive history, parity, gravidity, pre-pregnancy body mass index (BMI), weight gain of the mother during pregnancy.

Concerning the neonatal period the following data are collected: exposure to iodine excess (use of iodine-containing antiseptics), health problems of the newborn, type of delivery, season of birth and perinatal anoxia.

Concerning the period between birth and the home visit the following information is gathered about the parents: maternal/paternal education and employment, household income, marital status, area of residence, maternal age and information about housing.

Concerning the period between birth and the home visit the following information is gathered about the child: breastfeeding, chronic disease, attending nursery school, bilingualism, previous intellectual assessment.

In addition a self-report questionnaire is used to assess several psychological factors supposed to influence mental development: child’s negative life events, maternal mental health, maternal social support, marital discord and parent–child interactions. Some of the questions were adapted from existing questionnaires [80-83].

## Discussion and conclusion

Several studies showed that elevated TSH levels after birth were associated with suboptimal cognitive and psychomotor outcomes [12-15]. However, many factors may influence neonatal TSH [16-27] concentration and some of them may affect both TSH levels and neurodevelopment in childhood [14,23,28-59]. The present study aims to evaluate association between elevated TSH at birth and intellectual, psychomotor and psychosocial development of preschool children taking into account confounding factors. This study aims to clarify the effect of MID during pregnancy on the neurodevelopment of the offspring. Therefore, the results may have important implications for future public health recommendations, policies and practices in food supplementation.

In order to evaluate the efficiency of national programmes implemented to suppress iodine deficiency, adapted surveillance of the iodine status of the population is needed. To monitor the iodine status of a population, neonatal TSH whole blood concentration can be used as an indicator [60]. The proposed cut-off percentage below 3% of the TSH results >5 mU/L was shown to be not sensitive enough to detect MID [27,72]. The present study aims to reassess the recommended cut-off of >5 mU/L using ROC curve analysis. Therefore, the results of the study could help to clarify the potential use of neonatal TSH screening results for monitoring the iodine status of populations. Furthermore, the results could lead to the definition of a new neonatal TSH cut-off for monitoring the iodine status of populations. The results could also have an impact on the definition of the cut-off of TSH levels used for reporting by neonatal screening centres.

## Abbreviations

MID: Mild iodine deficiency; ROC: Receiver operating characteristic; TH: Thyroid hormone; TRH: Thyrotropin-releasing hormone; TSH: Thyroid-stimulating hormone; WHO: World Health Organization.

## Competing interest

All authors declare not having any conflict of interest with regard to this study.
